# Supplementary material for: Scalable and efficient separation of hydrogen isotopes using graphene-based electrochemical pumping
Source: Nat Commun. 2017 May 9;8:15215. doi: 10.1038/ncomms15215 (PMC5436102; doi:10.1038/ncomms15215)
Supplement: Supplementary Information — Supplementary Figures, Supplementary Notes and Supplementary References [file ncomms15215-s1.pdf]

Supplementary information

Supplementary Figures

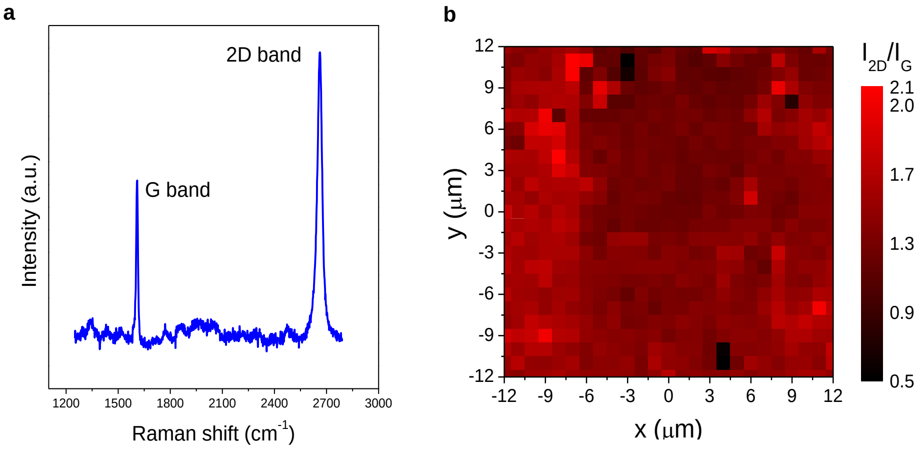

Supplementary Figure 1| Raman spectroscopy. a, Raman spectrum of CVD graphene on Nafion with the background subtracted. **b**, 2D/G Raman map over a 24μm × 24μm area.

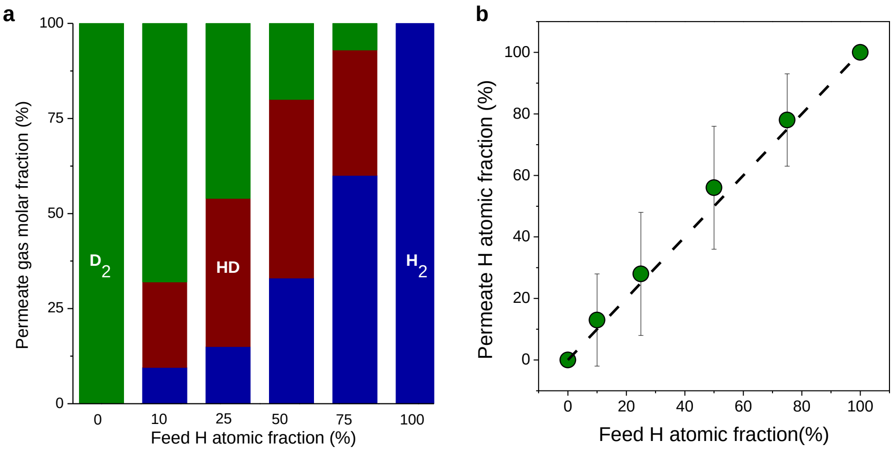

Supplementary Figure 2| Mass spectrometry measurements with control devices. a, Molar fractions of gases in the permeate for six different protium fractions in the feed for one of the devices. b, Permeate protium fraction for the same six feed protium fractions; average of two devices is shown.

Supplementary Notes

Transfer characterization

If desired, it is possible to prepare membranes without carbon cloth, for example, to allow for optical examination of the transferred graphene. We also characterized such films with SEM and Raman spectroscopy. The Raman spectrum revealed the absence of a D peak and a 2D/G ratio of ≈2 over most of the sample. This confirmed that our transfer method did not significantly introduce microscopic defects into the films (see Supplementary Figure 1).

Mass spectrometry measurements

We note that, in general, if the molar fractions in the feed are known, the atomic fraction of protium (and hence of deuterium) in the output can be readily calculated. Because a D_2 molecule consists of two D atoms, HD of one H atom and one D atom and H_2 of two H atoms, the fraction of protium atoms [H] in the permeate is:

$$[H] = \{(^1/_2)[HD]+[H_2]\}/\{[D_2]+[HD]+[H_2]\} \qquad (1)$$

An equivalent relation is also true for the deuterium fraction in the permeate:

$$[D] = \{(^1/_2)[HD]+[D_2]\}/\{[D_2]+[HD]+[H_2]\} \qquad (2)$$

Using these equations we extracted the permeate H atomic fractions shown in Figure 2 of the main text.

To verify our mass spectrometry results, we measured two reference devices without graphene; that is, a Nafion membrane sandwiched between two carbon cloth electrodes (with catalysts present on both carbon cloth electrodes). Supplementary Figure 2 shows our results. As expected, no isotope separation was observed with these devices. It is relevant to mention that, in principle, even in the absence of graphene it is possible to observe a difference in the production of protium over deuterium due to different rates of gas evolution for different isotopes at the electrodes[1-6]. We did not detect this effect in our control experiments without graphene (Supplementary Figure 2), in agreement with a previous report[7]. This is not surprising for two reasons. First, the isotope effect reported using Nafion as the polymer electrolyte is small, with $\alpha \approx 3$ even under optimum conditions[2]. Such a small α would result in differences in permeate H atomic fractions within the error bars of Supplementary Figure 2. Second, to achieve the above small separation factor, the electrodes need to be preconditioned and, due to the sensitivity of electrolysis to applied voltages and current densities, the latter parameters must be carefully selected[1-6]. These considerations show that, besides providing a large separation factor, graphene membranes are a very robust isotope separation technology.

Energy estimations

An important parameter to consider in any separation process is the amount of feed that must be processed during the separation. This can be estimated using the Rayleigh equation[8]:

$$\ln(W_0/W) = (\alpha-1)^{-1}\{\ln(x_{W0}/x_W)+\alpha\ln[(1-x_W)/(1-x_{W0})]\} \qquad (3)$$

where W_0 is the number of moles of initial feed, W the number of moles remaining at the end of the enrichment process, x_{W0} the mole fraction of the light isotope in the initial feed and x_W the mole fraction of the light isotope in the final product. Using this equation we estimate that to enrich a protium-deuterium mixture using graphene ($\alpha \approx 8$) from 0.015% (natural deuterium abundance) up to a 20% deuterium purity we would need to process ≈3785 moles of feed per mole of product. The power density ($p=P/A$) per flux unit is given by $p/\Phi = P/(A\Phi) = IV/A\Phi$. But from Faraday's law, $\Phi = I/2FA$, therefore $p/\Phi = 2FV$ and depends only on the operating voltage and Faraday's constant. Operating the graphene electrochemical pump at 0.5V would therefore require ~371 MJ mol^{-1}. This is equivalent to ~20 GJ per kg of 20% enriched heavy water.

It is instructive to examine the effect of raising the temperature in our membranes. On the one hand, the proton and deuteron transport through graphene is an Arrhenius activated process[7]. Therefore, if we increase the operating temperature, the ratio of conductance across the membrane at high and room temperature would be given by: $S_H/S_L \approx \exp[-\Delta E(1/kT_H - 1/kT_L)]$ where ΔE is the activation energy of the process, k is Boltzmann's constant, T_H is the high temperature, T_L is room temperature and S_H and S_L are the high and room temperature conductance, respectively. Raising the temperature to ≈60°C, we get $S_H/S_L \sim 10$. From this increase in membrane conductance (decrease in resistance) we can estimate a reduction in the operating voltage by the same factor for a given Φ. On the other hand, increasing the temperature should reduce the proton-deuteron separation factor. Indeed, for monocrystalline graphene it is given by $\alpha = \exp(60\text{meV}/kT) \approx 10$, therefore at ≈60°C it is $\alpha \approx 8.5$. In our CVD membranes, we find a smaller room temperature separation factor of $\alpha \approx 8$. Therefore, assuming a reduction similar as in monocrystalline graphene, we can expect a separation factor of $\alpha \approx 7$. This drop in separation impacts the amount of feed that needs to be processed which goes from ≈3847 moles to ≈4591 moles – just 20% more. So, using the same formula as in the last paragraph to calculate the power supplied, we can now estimate that it would require ~2 GJ per kg of enriched heavy water. In the above estimation we have neglected the cost of the heat energy supplied to the system for the following reason. We note that, like the NH_3/H_2 exchange processes[9], graphene membranes use hydrogen gas as feed which contains ~221 kJ mol^{-1}; much more than the energy necessary to heat a diatomic gas by 30°C: $\Delta Q = {}^5/_2\, R\, \Delta T \approx 0.6$ kJ mol^{-1}.

Using hBN could also reduce operational energy costs. To incorporate this material into our electrochemical pumps we would need to use it in combination with a nm-thick Pd film to provide the necessary electrical conductivity. Since Pd is itself a hydrogen-deuterium selective material[10,11] (room temperature $\alpha \approx 2$) and it would only be in direct contact with Nafion over ~5% of the membrane area, we don't expect significant drops in separation factor associated with leakage through cracks in the hBN films. As for throughput across hBN-Pd membranes, we note that Pd membranes are highly permeable to hydrogen; even micrometer thick membranes can produce a hydrogen flux of the order of ~1 mmol cm^{-2} h^{-1} at room temperature[11,12]. Therefore, the incorporation of Pd into hBN could prove a promising technology. Yet another approach to reduce the operational energy of the system would be to increase the area of the membrane for a fixed gas flux. Indeed, the total throughput is given by ΦA. As discussed above, reducing V reduces Φ but proportionally increasing A keeps the total throughput constant.

Supplementary References

1. Conway, B. E. The Electrolytic Hydrogen-Deuterium Separation Factor and Reaction Mechanism. *Proc. R. Soc. A Math. Phys. Eng. Sci.* **247,** 400–419 (1958).
2. Ivanchuk, O. & Goryanina, V. Isotopic effects of hydrogen during the decomposition of water in electrolysis with a solid polymer electrolyte. *At. Energy* **89,** 229–233 (2000).
3. Walton, B. Y. H. F. The electrolytic separation of deuterium; the influence of temperature and current density at various cathode metals. *Trans. Faraday Soc.* **34,** 436–449 (1938).
4. Roy, P. Influence of Temperature on the Electroytic Separation Factor Of Hydrogen Isotopes. *Can. J. Chem.* **40,** 1452–1460 (1962).
5. Fukuda, M. & Horiuti, J. Separation Factor of Deuterium on Platinum Hydrogen Electrode. *J. Res. Inst. Catal.* **10,** 43–72 (1962).
6. Lewis, G. P. & Ruetschi, P. The dependence of the electrolytic hydrogen-deuterium separation factor on the electrode potential. *J. Phys. Chem.* **66,** 1487–1492 (1962).
7. Lozada-Hidalgo, M. *et al.* Sieving hydrogen isotopes through two-dimensional crystals. *Science* **351,** 68–70 (2016).
8. Gorak, A. & Sorensen, E. *Distillation: Fundamentals And Principles*. (Elsevier, 2014).
9. Miller, A. I. Heavy Water: A Manufacturers' Guide for the Hydrogen Century. *Can. Nucl. Soc. Bull.* **22,** 1–14 (2001).
10. Nishikawa, M., Shiraishi, T. & Murakami, K. Solubility and Separation Factor of Protium-Deuterium Binary Component System in Palladium. *J. Nucl. Sci. Technol.* **33,** 504–510 (1996).
11. Lu, G. Q. *et al.* Inorganic membranes for hydrogen production and purification: A critical review and perspective. *J. Colloid Interface Sci.* **314,** 589–603 (2007).
12. Uemiya, S., Matsuda, T. & Kikuchi, E. Hydrogen permeable palladium-silver alloy membrane supported on porous ceramics. *J. Memb. Sci.* **56,** 315–325 (1991).
